# [^11^C]carfentanil PET imaging for studying the peripheral opioid system in vivo: effect of photoperiod on mu-opioid receptor availability in brown adipose tissue

**DOI:** 10.1007/s00259-022-05969-5

**Published:** 2022-09-27

**Authors:** Lihua Sun, Richard Aarnio, Erika Atencio Herre, Salli Kärnä, Senthil Palani, Helena Virtanen, Heidi Liljenbäck, Jenni Virta, Aake Honkaniemi, Vesa Oikonen, Chunlei Han, Sanna Laurila, Marco Bucci, Semi Helin, Emrah Yatkin, Lauri Nummenmaa, Pirjo Nuutila, Jing Tang, Anne Roivainen

**Affiliations:** 1 Department of Nuclear Medicine, Huashan Hospital, Fudan University, Shanghai, China; 2 Turku PET Centre, University of Turku and Turku University Hospital, 20520 Turku, Finland; 3 Turku Center for Disease Modeling, University of Turku, 20520 Turku, Finland; 4 Heart Center, Turku University Hospital, 20520 Turku, Finland; 5 Division of Clinical Geriatrics, Center for Alzheimer Research, Department of Neurobiology, Care Sciences and Society, Karolinska Institute, 17177 Stockholm, Sweden; 6 Theme Inflammation and Aging, Karolinska University Hospital, 14186 Stockholm, Sweden; 7 Turku PET Centre, Åbo Akademi University, Turku, Finland; 8 Central Animal Laboratory, University of Turku, 20520 Turku, Finland; 9 Department of Psychology, University of Turku, 20520 Turku, Finland; 10 Department of Endocrinology, Turku University Hospital, 20520 Turku, Finland; 11 InFLAMES Research Flagship Center, University of Turku, 20520 Turku, Finland; 12 Research Program in Systems Oncology, Faculty of Medicine, University of Helsinki, 00014 Helsinki, Finland

**Keywords:** Brown adipose tissue, Carfentanil, Mu-opioid receptor, Photoperiod, Positron emission tomography

## Abstract

**Purpose:**

Photoperiod determines the metabolic activity of brown adipose tissue (BAT) and affects the food intake and body mass of mammals. Sympathetic innervation of the BAT controls thermogenesis and facilitates physiological adaption to seasonal changes, but the exact mechanism remains elusive. Previous studies have shown that central opioid signaling regulates BAT thermogenesis, and that the expression of the brain mu-opioid receptor (MOR) varies seasonally. Therefore, it is important to know whether MOR expression in BAT shows seasonal variation.

**Methods:**

We determined the effect of photoperiod on BAT MOR availability using [^11^C]carfentanil positron emission tomography (PET). Adult rats (*n* = 9) were repeatedly imaged under various photoperiods in order to simulate seasonal changes.

**Results:**

Long photoperiod was associated with low MOR expression in BAT (*β* =  − 0.04, 95% confidence interval: − 0.07, − 0.01), but not in muscles. We confirmed the expression of MOR in BAT and muscle using immunofluorescence staining.

**Conclusion:**

Photoperiod affects MOR availability in BAT. Sympathetic innervation of BAT may influence thermogenesis via the peripheral MOR system. The present study supports the utility of [^11^C]carfentanil PET to study the peripheral MOR system.

**Supplementary Information:**

The online version contains supplementary material available at 10.1007/s00259-022-05969-5.

## Introduction

The well-characterized role of brown adipose tissue (BAT) is to rapidly produce heat through nonshivering thermogenesis, and this demonstrates a seasonal rhythm. Environmental factors, such as photoperiod, determine the metabolic activity of BAT. Short photoperiod markedly stimulates BAT growth and thermogenesis in Syrian hamsters, along with greater food intake and weight gain [1]. This effect of photoperiod on BAT thermogenesis seems to be independent of the parallel effect of environmental temperature variation [2, 3], suggesting that a distinct physiological pathway is responsible for this seasonal rhythm. However, the exact neurochemical determinants of the seasonal pattern of BAT function remain elusive.

The endogenous opioid system regulates pain, stress, and emotions [4], and in particular, central mu-opioid receptor (MOR) signaling is a potent regulator of feeding [5–7]. We have recently shown that photoperiod drives the seasonal pattern of central MOR expression [8]. In contrast to central MOR signaling, understanding of the roles of the peripheral opioid system is largely based on the results of studies of pain, as exemplified by the local analgesic effect of opioid peptides [9]. MOR expression has been also demonstrated in immune cells [10]; tissue injury augments peripheral opioid analgesia alongside an increase in MOR expression [11, 12], suggesting that MOR signaling plays a role in the immune system and inflammation. In mammals, MORs are also expressed in peripheral tissues, such as muscle, intestine, adrenal, kidney, lung, and liver [13, 14]. However, knowledge of the roles of peripheral opioid receptors is limited, and to the best of our knowledge, no previous studies have determined whether MORs are expressed in BAT.

BAT is innervated by a large number of sympathetic nerves, which control thermogenesis [15, 16]. A role of opioid signaling as a modulator of BAT thermogenesis is supported by data showing that intravenous or intracerebroventricular administration of fentanyl increases BAT sympathetic nerve activity and thermogenesis [17]. There have been no studies of the role of peripheral MOR signaling in BAT activation. However, the inflammatory response in BAT has been shown to be associated with lower thermogenic capacity [18], and inflammation is associated with greater opioid signaling [9, 11], which suggests a possible effect of peripheral MOR on BAT activity.

In the present study, we aimed to determine whether peripheral MOR signaling is involved in the effects of photoperiod on BAT activity. Rats were kept under various photoperiods to simulate seasonal cycles, and each was repeatedly imaged using [^11^C]carfentanil PET. Using the data obtained, we previously showed that photoperiod influences in vivo brain MOR expression [8]. In addition, we showed that seasonal changes in photoperiod affect stress hormone concentrations and weight gain. Here, we further studied the effect of photoperiod on the expression of MOR in BAT. We hypothesized that photoperiod would influence BAT function via the MOR signaling pathway.

## Materials and methods

### Animal handling and seasonal simulation

Eighteen adult Sprague–Dawley rats (Envigo, Horst, Netherlands; age > 90 days; 11 males, 7 females) were housed under controlled laboratory conditions in open-top cages with free access to water and CRM-E diet (SDS, Witham, UK). Rats were caged in groups of two or three same-sex individuals. The study comprised an experimental group (nine males, five females), for which the lighting duration was programmed, such that the photoperiod changed over an abbreviated cycle of 3 months, and a control group (two males, two females), which was under a constant photoperiod (12-h light/dark cycle), with all other conditions being the same as for the experimental group. The control group was used to account for the potential effect of aging on MOR expression, and the statistical analyses pertaining to MOR expression were also separately conducted in the experimental group. The same type of LED lighting was used for the control and experimental groups. The study was conducted in accordance with EU Directive 2010/63/EU regarding the protection of animals used for scientific purposes, based on the principle of the 3Rs, and all procedures and protocols were approved by the National Project Authorization Board of Finland (license numbers ESAVI/3116/04.10.07/2017 and ESAVI/8648/2020).

### PET imaging and processing

**Fig. 1:**
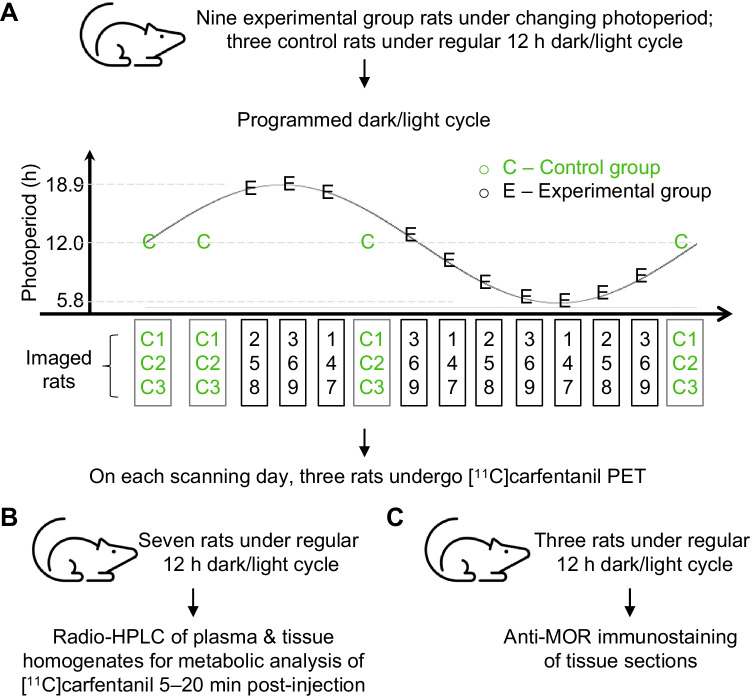
Experimental study design. The study includes **A** repeated [^11^C]carfentanil PET imaging, **B** radio-HPLC analysis, and **C** immunofluorescence staining. HPLC, high-performance liquid chromatograph

Twelve of eighteen rats (experimental group: six males, three females; control group: two males, one female) were studied using 60-min dynamic [^11^C]carfentanil PET imaging on three to four occasions under isoflurane anesthesia (Fig. 1A). A CT scan was always performed for anatomical reference and attenuation correction. Rats in the control and experimental groups were scanned following a similar PET protocol. The radiotracer was divided among three rats (two males, one female) and two different scanners on each scanning day to maximize the data collection per batch. Because of their larger size, an Inveon Multimodality PET/CT scanner (Siemens Medical Solutions, Knoxville, TN, USA) was used to image the male rats (two rats at a time), whereas a Molecubes PET/CT scanner (Gent, Belgium) was used for the female rats. In total, 42 PET/CT scans were performed. The rats were weighed on the scanning day, and then for the Inveon Multimodality PET/CT scanner, which had lower resolution and sensitivity, a 5 MBq dose of [^11^C]carfentanil (actual 4.69 ± 0.60 MBq), corresponding to 29.28 ± 20.53 ng/kg, was injected. For the Molecubes PET/CT scanner, 1 MBq (actual 1.14 ± 0.13 MBq), corresponding to 13.92 ± 11.74 ng/kg, was injected. While there may have been receptor saturation, the saturation would have been similar under all the test conditions, and therefore, this should not have affected comparisons between conditions. Accordingly, the effects of sex and dose cannot be separated from the scanner type or dose-dependent effects.

Dynamic PET images were analyzed using Carimas software (version 2.10.3.0), which was developed at the Turku PET Centre, Finland. The PET datasets were reconstructed into 20 time frames using the three-dimensional ordered subset expectation maximization (OSEM3D) algorithm: 6 × 0.5 min, 3 × 1 min, 4 × 3 min, and 7 × 6 min. PET and CT images were automatically superimposed and visually inspected by researchers. Regions of interests (ROIs) were defined based on CT image independently by two researchers (HV and EAH) who were blinded to the experimental conditions.

### Analysis of the fate of the injected [^11^C]carfentanil

Four female and three male rats that were not involved in the PET imaging study, and kept under a regular 12-h dark/light cycle, were anesthetized using isoflurane and injected intravenously with [^11^C]carfentanil (39.5 ± 17.5 MBq) to assess the in vivo stability of the tracer in plasma and tissues (Fig. 1B; Supplementary Fig. S1). Blood samples (0.2–0.6 mL) were drawn using cut-tail method 5 and 10 min after injection. Twenty minutes after injection, blood was collected by cardiac puncture, and the rats were euthanized by cervical dislocation. Blood samples were collected into heparinized gel tubes (Microtainer, Becton, Dickinson and Company, Franklin Lakes, NJ, USA) and centrifuged at 14,400 × *g* for 90 s. The separated plasma was mixed with ice-cold acetonitrile (plasma:acetonitrile 2:3 (*v*/*v*)) to precipitate the plasma proteins and then centrifuged at 14,400 × *g* for 90 s. The supernatants were analyzed using a high-performance liquid chromatograph coupled to a radiodetector (radio-HPLC: Merck Hitachi L-7100 gradient pump system with Radiomatic 150 TR, Packard, Palo Alto, CA, USA). The brain, BAT, liver, stomach, muscle, and duodenum were dissected and homogenized in 1:1 (*v*/*v*) methanol:water using an electric homogenizer (Ultra-Turrax T8, IKA, Staufen, Germany). The homogenates were filtered (Amicon Ultra-4, 50 k, Merck KGaA, Darmstadt, Germany) to remove particulate matter and proteins, and then injected into the radio-HPLC. In the radio-HPLC, a µBondapak C18 column (7.8 × 300 mm, 10 µm; Waters, Milford, MA, USA) was used, along with a gradient of 50 mM phosphoric acid and acetonitrile, and the results were analyzed as previously described [19].

### Modeling of MOR availability

Standardized uptake values (SUVs) were calculated by dividing tissue radioactivity concentrations (MBq/mL) by the injected radioactivity dose per unit animal mass (MBq/g). One approach used to estimate the MOR availability was to calculate the SUV ratios for the areas under the time-activity curves (TACs) for BAT and muscle (the reference tissue) because this area represents an approximate index of MOR availability. On the basis of our initial radiometabolite analysis, the first 5 min (because of confounding effects of perfusion in the periphery) and the last 40 min (because of radiotracer decomposition) of the TACs were excluded from this modeling.

### Immunofluorescence staining

Brain, BAT, muscle, stomach, and white adipose tissue from healthy male Sprague–Dawley rats (*n* = 3, kept under a regular 12-h dark/light cycle and not involved in the PET imaging study, Fig. 1C) were collected on ice, frozen, and cryosectioned at 10 µm thickness (transverse sections of brain were prepared).

Tissue sections were fixed in 10% formalin for 10 min, permeabilized in 0.1% Triton X-100 in phosphate-buffered saline (PBS) for 5 min, and washed between and after these incubations with PBS. To block nonspecific staining, sections were incubated in 5% normal goat serum (NGS; Vector Laboratories, Burlingame, CA, USA) in PBS for 1 h at room temperature. Then, the sections were incubated with anti-OPRM1 (1:100 dilution with 2.5% NGS in PBS, #MAB8629, Bio-Techne, Minneapolis, MN, USA) overnight at 4 °C. After PBS washes, the sections were incubated with AlexaFluor 488-conjugated anti-rabbit secondary antibody (1:500 dilution with 2.5% NGS in PBS, Invitrogen, Waltham, MA, USA) for 1 h at room temperature, followed by staining with 4′,6-diamidino-2-phenylindole (DAPI, 1:10,000 dilution with MilliQ water; Sigma-Aldrich, St. Louis, MO, USA) for 10 min. Finally, the sections were mounted using Prolong Gold Antifade mounting media (#P369309, Thermo Fisher Scientific, Waltham, MA, USA). Sections that had not been incubated in primary antibody were used to confirm the specificity of the staining. Images were obtained using a 3i Spinning Disk confocal microscope and Slidebook software (Intelligent Imaging Innovations, Denver, CO, USA), and processed using ImageJ software (NIH, Bethesda, MD, USA).

### Statistical analysis

Repeated measurements were analyzed using a linear mixed-effects model, with varying intercepts for each rat and fixed-effect factors, such as age, photoperiod, and scanner type. R statistical software (version 4.0.3; r-project.org) with the lme4 package was used.

## Results

### Peripheral MOR expression and [^11^C]carfentanil PET imaging

**Fig. 2:**
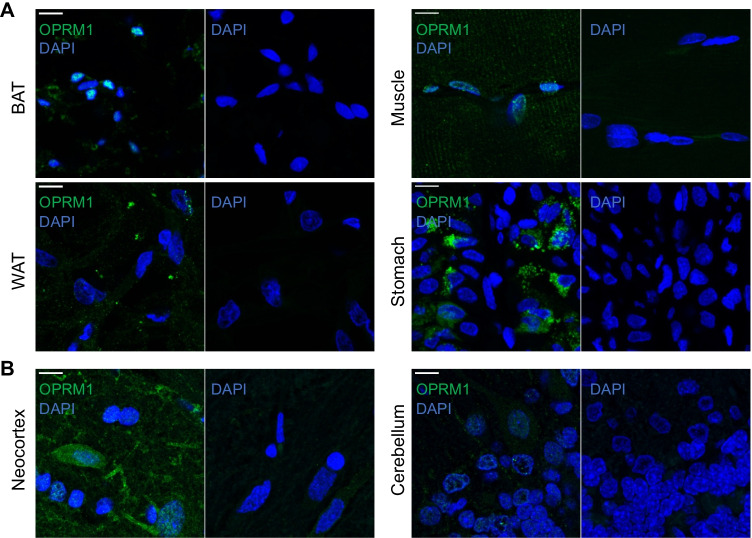
Representative immunofluorescence confocal microscopy images obtained using anti-MOR antibody (OPMR1, green) reveals expression in **A** peripheral tissues and **B** brain regions in the left panels. Nuclei were counterstained with 4′,6-diamidino-2-phenylindole (blue). Omission of the anti-MOR antibody resulted in no green staining, as shown in the right panels. BAT, brown adipose tissue; WAT, white adipose tissue. Scale bar = 10 µm

Immunofluorescence staining showed the highest expression of MOR in the BAT and stomach (Fig. 2A), with lower expression in muscle and almost no expression in white adipose tissue. Consistent with published data [20], MOR was expressed at a higher level in the neocortex than in the cerebellum (Fig. 2B).

**Fig. 3:**
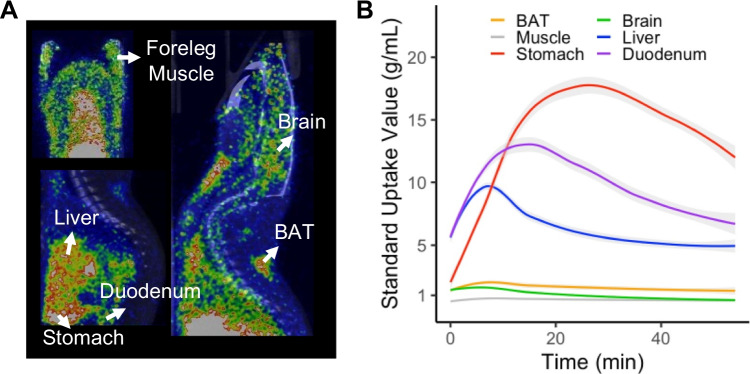
[^11^C]carfentanil standardized update values (SUVs) in various tissues. **A** Positron emission tomography/computed tomography fusion image of a rat, with the regions of interest labeled. **B** Regional time-activity curves for the SUVs during the scans. Shaded areas represent 95% confidence intervals

To analyze the PET images, we first calculated SUVs for the brain and five peripheral tissues (BAT, muscle, stomach, liver, and duodenum) (Fig. 3A) using the TACs derived from dynamic PET images (Fig. 3B). The SUVs of the stomach, liver, and duodenum were higher than those of the BAT, brain, and muscle.

### Tissue-specific decomposition of [^11^C]carfentanil

To rule out nonspecific binding of radioactive metabolites, we studied the metabolic profiles of [^11^C]carfentanil in various tissues. Analysis of blood samples demonstrated continuous decomposition of the radiotracer (Table 1). Measurements made in seven rats confirmed that, 20 min after radiotracer injection, the SUVs of the liver and duodenum were largely determined by the uptake of radioactive metabolites. Therefore, [^11^C]carfentanil PET is not the optimal tool for the study of MOR availability in these tissues. Comparable [^11^C]carfentanil radiometabolism profiles were found for BAT, muscle, and stomach, with [^11^C]carfentanil contributing over 50% of the total radioactivity 20 min after radiotracer injection. The brain showed the highest proportion of intact [^11^C]carfentanil.

**Table 1:** Percentage of intact [^11^C]carfentanil in rat tissues at different time points after intravenous injection

Time after injection	Blood	BAT	Muscle	Brain	Stomach	Liver	Duodenum
5 min	74.40 ± 10.97	ND	ND	ND	ND	ND	ND
10 min	57.18 ± 12.61	ND	ND	ND	ND	ND	ND
20 min	44.53 ± 9.23	66.49 ± 14.05	60.54 ± 9.30	75.63 ± 7.37	50.04 ± 10.80	5.40 ± 4.08	7.89 ± 3.54

Results are expressed as mean ± standard deviation (*n* = 7). *BAT*, brown adipose tissue; *ND*, not determined

In the stomach, approximately 50% of the [^11^C]carfentanil remained 20 min after tracer injection, and the SUV showed a much higher level of MOR availability. However, the stomach was not scanned in some of the images, and therefore, the stomach data were not further analyzed.

### Effect of photoperiod on [^11^C]carfentanil uptake

**Fig. 4:**
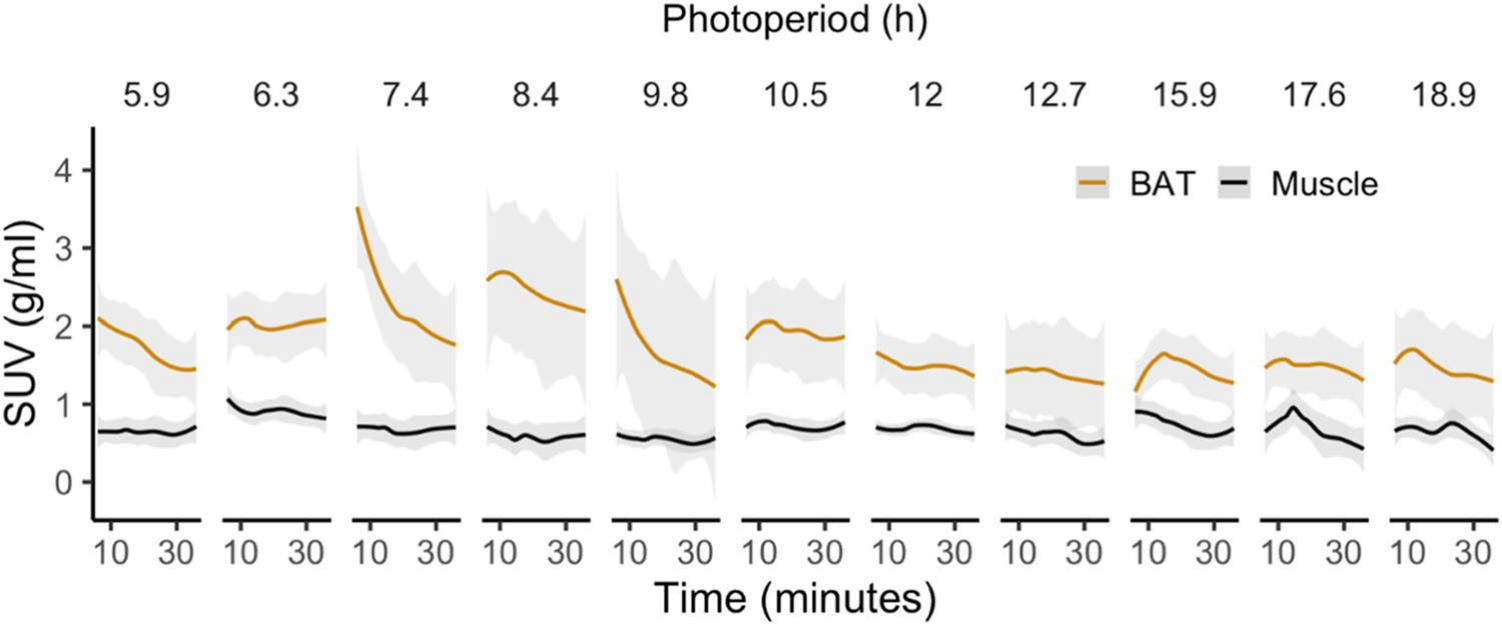
Time-activity curves (TACs) for brown adipose tissue and muscle during the various photoperiods. Standardized uptake values (SUV) between 5 and 40 min are shown. Shaded areas represent 95% confidence intervals

TACs for [^11^C]carfentanil SUVs in BAT and muscle were plotted according to photoperiod (Fig. 4), and we found that the areas under the TACs for BAT were smaller when the photoperiod was long. BAT showed greater radiotracer uptake than muscle on all the scans.

### Effect of photoperiod on MOR availability in BAT

The SUVs (Fig. 3B) and metabolic profiles of [^11^C]carfentanil were comparable in BAT and muscle; therefore, the BAT-to-muscle SUV ratio was used to estimate the availability of MOR in BAT.

To analyze MOR availability, we compared both the experimental group (*n* = 3) and control group (*n* = 9) using three statistical models: (i) model 1, with scanner type as the only fixed-effect factor; (ii) model 2, with scanner type and photoperiod as fixed-effect factors; and (iii) model 3, with scanner type, photoperiod, and age as fixed-effect factors. In all these mixed-effect models, rat identity was used as a random factor. The results showed that model 2 was the best model, with the lowest Akaike information criterion (AIC) value (model 1: 71.4; model 2: 67.9; model 3: 69.9) and the largest marginal *R*^2^ (model 1: 0.09; model 2: 0.18; model 3: 0.17). Model 2 showed that photoperiod had a significant effect on MOR availability in BAT (*β* =  − 0.04, 95% confidence interval (CI) − 0.08, − 0.01). Model 3 showed that age had no effect (*β* =  − 0.0002, 95% CI: − 0.006, 0.006) on MOR availability, and therefore, age was dropped as a factor in subsequent analyses.

Statistical analysis was also performed using only the experimental group (*n* = 9), which was exposed to changing photoperiod. The MOR availability of BAT was analyzed using the fixed-effect factors of scanner type and photoperiod, and the random factor of rat identity. Consistent with the above finding, increasing daylength was associated with a reduction in peripheral MOR binding in BAT (*β* =  − 0.04, 95% CI: − 0.07, − 0.01). A plot of the SUV ratios (Supplementary Fig. S2A) demonstrated that photoperiod was inversely associated with MOR availability in BAT.

### Comparison of the BAT and muscle SUVs

In addition, we analyzed the [^11^C]carfentanil SUVs separately for BAT and muscle. The area under the TAC (5–20 min) was used as an index of the availability of MOR. When both the control and experimental groups were analyzed, increasing photoperiod was associated with lower MOR availability in BAT (*β* =  − 0.037, 95% CI: − 0.07, − 0.01) but not in muscle (*β* = 0.005, 95% CI: − 0.01, 0.02). When the experimental group was analyzed alone, similar results were obtained for BAT (*β* =  − 0.04, 95% CI: − 0.06, − 0.01) and muscle (*β* = 0.006, 95% CI: − 0.01, 0.02). Plots of the SUVs (Supplementary Fig. S2B) showed that photoperiod had an inverse relationship with MOR availability in BAT, but not in muscle.

## Discussion

The principal finding of the present study is that photoperiod affects the availability of MOR in BAT, with longer photoperiod being associated with lower availability of MOR. The lower MOR availability in BAT during long days may be the cause of lower BAT thermogenesis, which is consistent with the lower BAT thermogenesis that occurs during summer [1, 3]. The neurotransmitter signaling that regulates BAT metabolism is poorly understood, but the present data suggest that MOR signaling is an important regulator of BAT activity. When taken together with our previous findings regarding the effects of photoperiod on the central MOR system [8], it seems that both central and peripheral MOR signaling pathways are activated in a seasonal pattern.

The present study also demonstrates the utility of [^11^C]carfentanil PET for the study of peripheral MOR signaling in vivo. Previous PET imaging studies have exclusively focused on the central opioid signaling, and to our knowledge, this is the first to extend the application of in vivo PET measures to the assessment of peripheral MOR system. While opioid signaling is not confined in the central nervous system, a better understanding of its function in the peripheral regions is demanded.

### Potential role of MOR signaling in BAT functions

The exact mechanism whereby photoperiod affects MOR availability in BAT remains to be determined. However, the MOR signaling pathway may be responsible for transmitting signals as part of the gut-brain-BAT axis. Recent studies have shown that BAT may have roles beyond the known pattern thermogenesis, and have supported the existence of a gut-BAT-brain axis that tunes neurometabolic control and eating behavior [21–23]. MORs are expressed throughout the central and enteric nervous systems [24], and have effects on gastrointestinal motility and possibly on feeding behavior [25]. In addition, the activation of central MOR signaling by fentanyl has been shown to induce BAT thermogenesis [17, 26]. Although previous studies have in general focused on regulatory signals from the brain to the BAT, afferent feedback from BAT to the brain has also been proposed [21, 23]. No evidence has been collected to date regarding a potential role of MOR in this; however, in the present study, we have shown that photoperiod not only affects central MOR availability, but also receptor availability in BAT. This suggests a potential novel means of communication between the BAT and brain. Furthermore, low MOR availability in BAT may imply a lower level of communication between the brain and BAT during the lighter seasons.

Central MOR signaling contributes to hedonic or “liking” responses in the brain [27], which are related to the food-reward response [5]. Obese individuals have low brain MOR expression, and bariatric surgery normalizes this expression [28], which further implies a role for central MOR in feeding behavior and weight gain. Despite of the well-characterized roles of MOR signaling in the central nervous system and behavior, how this signaling pathway functions beyond the nervous system remains elusive. Here, our data showing that MOR availability in BAT demonstrates seasonal patterns highlight its potential endocrine and paracrine role. Along with PET findings, immunofluorescence staining data showed high expression of MOR in peripheral tissues, consistent with the results of prior studies [13, 14]. In particular, we found that MOR is highly expressed in BAT.

Our finding that photoperiod affects MOR availability may also be explained by greater peripheral opioid release, resulting in greater competition with the radiotracer for MOR binding during longer days. It has been hypothesized that longer photoperiod drives skin cells to secrete endogenous opioids therefore to affect mood and behavior [29, 30]. Peripheral opioid signaling is also activated during inflammatory responses because of the secretion of endogenous opioid by immune cells [10, 11, 31]. An inflammatory response in BAT leads to reductions in energy expenditure and glucose uptake [18], which is consistent with the lower thermogenesis in BAT during long days [1, 3]. However, no direct evidence for this mechanism is provided by the present study.

### [^11^C]carfentanil PET to study the peripheral opioid system

In the present study, we have shown that [^11^C]carfentanil PET can be used to assess MOR availability in peripheral tissues in which the accumulation of radioactive metabolites of [^11^C]carfentanil is not substantial. Compared to organs such as the liver and duodenum, decomposition of the radiotracer or accumulation of radioactive metabolites in BAT, muscle, and brain is significantly lower. The current finding extends the application of [^11^C]carfentanil PET from being restricted in brain measures to assessment of peripheral tissues. Although gastric MOR function was not assessed in the present study, the data suggest that the effects of MOR signaling on gastric function and eating behavior should be studied in vivo in the future.

MOR signaling in BAT may be important for energy homeostasis and eating behavior in humans, while the functional BAT in adults has become a new target for anti-obesity and anti-diabetic therapies focusing on increasing energy expenditure. The findings of this study revealing an active MOR signaling pathway in BAT may have potential clinical benefits in the future treatment of obesity. Because the body mass-based doses of [^11^C]carfentanil administered to the rats are comparable to those used in routine clinical imaging studies [19], [^11^C]carfentanil PET could also be used to study peripheral MOR function in humans.

## Limitations

Although we have shown that photoperiod affects MOR signaling in the brain and BAT, the pattern of regulation is unclear (for example, an inverse linear vs. an inverted-U relationship). Brain and BAT showed comparable MOR availability (see Figs. 2 and 3), but much larger amounts of MOR were found in other peripheral tissues. For instance, the stomach showed much higher MOR availability than the BAT. We do not know how these relative densities affect endogenous opioid signaling and, therefore, eating behavior. Furthermore, we did not use magnetic resonance imaging to evaluate BAT mass, and therefore, we cannot rule out an effect of photoperiod on BAT volume [1], which would have affected [^11^C]carfentanil uptake. In addition, although we have shown that photoperiod is negatively associated with MOR availability in BAT, we do not know whether photoperiod-associated changes in MOR availability are reflected in changes in BAT metabolism and thermogenesis. Finally, the radiometabolism of [^11^C]carfentanil was studied in a relatively small number of rats (*n* = 7), and possible metabolic differences between the animals were not necessarily well revealed.

## Conclusion

We have shown that photoperiod affects the availability of MOR in BAT. This extends our previous finding that photoperiod affects MOR availability in the brain, and implies that there is a shared neurotransmitter signaling pathway in the brain and body that responds to the seasons. Seasonal affective disorders are characterized by winter depression and overeating. Considering the important roles of MOR signaling in emotion, eating behavior, and thermogenesis, and the important role of BAT in weight gain and the motivation to eat [21, 23], the present findings imply that the MOR signaling pathway may mediate seasonal affective changes. Finally, our data highlight the applicability of [^11^C]carfentanil PET in studying the peripheral MOR signaling.

## Supplementary Information

Below is the link to the electronic supplementary material.

Supplementary file1 (DOCX 637 KB)
