# Supplementary material for: [11C]carfentanil PET imaging for studying the peripheral opioid system in vivo: effect of photoperiod on mu-opioid receptor availability in brown adipose tissue
Source: Eur J Nucl Med Mol Imaging. 2022 Sep 27;50(2):266–74. doi: 10.1007/s00259-022-05969-5 (PMC9816189; doi:10.1007/s00259-022-05969-5)
Supplement: Supplementary file 1 — Supplementary file1 (DOCX 637 KB) [file 259_2022_5969_MOESM1_ESM.docx]

**Supplementary Data**

**[^11^C]carfentanil PET imaging for studying the peripheral opioid system in vivo: effect of photoperiod on mu-opioid receptor availability in brown adipose tissue**

**Running Head:** Seasonality in Peripheral Opioid System

Lihua Sun^1,2^, Richard Aarnio^2^, Erika Atencio Herre^2^, Salli Kärnä^2^, Senthil Palani^2^, Helena Virtanen^2^, Heidi Liljenbäck^2,3^, Jenni Virta^2^, Aake Honkaniemi^2^, Vesa Oikonen^2^, Chunlei Han^2^, Sanna Laurila^2,4^, Marco Bucci^2,5,6,7^, Semi Helin^2^, Emrah Yatkin^8^, Lauri Nummenmaa^2,9^, Pirjo Nuutila^2,10,11^, Jing Tang^12^, Anne Roivainen^2,3,11^

*^1^Department of Nuclear Medicine, Huashan Hospital, Fudan University, Shanghai, China; ^2^Turku PET Centre, University of Turku and Turku University Hospital, FI-20520 Turku, Finland; ^3^Turku Center for Disease Modeling, University of Turku, FI-20520 Turku, Finland; ^4^Heart Center, Turku University Hospital, FI-20520 Turku, Finland;^5^Division of Clinical Geriatrics, Center for Alzheimer Research, Department of Neurobiology, Care Sciences and Society, Karolinska Institute, SE-17177 Stockholm, Sweden; ^6^Theme Inflammation and Aging, Karolinska University Hospital, SE-14186 Stockholm, Sweden; ^7^Turku PET Centre, Åbo Akademi University, Turku, Finland; ^8^Central Animal Laboratory, University of Turku, FI-20520 Turku, Finland; ^9^Department of Psychology, University of Turku, FI-20520 Turku, Finland; ^10^Department of Endocrinology, Turku University Hospital, FI-20520 Turku, Finland; ^11^InFLAMES Research Flagship Center, University of Turku, FI-20520 Turku, Finland; ^12^Research Program in Systems Oncology, Faculty of Medicine, University of Helsinki, FI-00014 Helsinki, Finland;*

**FIGURE S1.** Metabolism of [^11^C]carfentanil in rat tissues after the intravenous injection of radiotracer. High-performance liquid chromatography measurements of the metabolism of [^11^C]carfentanil in (A) the blood and (B) peripheral organs. The measurements for a single rat are presented.

**FIGURE S2.** Plots of standardized uptake value (SUV) ratios and SUVs, demonstrating the effect of photoperiod on MOR availability. (A) Plot of the SUV ratios comparing BAT and muscle. (B) Separate plots of SUVs for brown adipose tissue (BAT) and muscle. Individual animals are represented by separate lines. The black dashed lines are the least-squares (LS) regression lines for the linear model, predicting the SUV ratios/SUVs for the entire group. Shaded areas represent the 95% confidence intervals for the LS curves.
